# *Acinetobacter baumannii* in Localised Cutaneous Mycobacteriosis in Falcons

**DOI:** 10.4061/2010/321797

**Published:** 2010-09-05

**Authors:** Margit Gabriele Muller, Ancy Rajeev George, Julia Walochnik

**Affiliations:** ^1^Abu Dhabi Falcon Hospital, EAD, P.O. Box 45553, Abu Dhabi, UAE; ^2^Abteilung Für Medizinische Parasitologie, Klinisches Institut für Hygiene und Medizinische Mikrobiologie, Medizinische Universität Wien, Kinderspitalgasse 15, 1095 Vienna, Austria

## Abstract

Between May 2007 and April 2009, 29 falcons with identically localized, yellowish discolored cutaneous lesions in the thigh and lateral body wall region were presented at Abu Dhabi Falcon Hospital. Out of 18 falcons integrated in this study, 16 tested positive to *Mycobacterium. avium* complex. The 2 negative falcons tested positive in the *Mycobacterium* genus PCR. Moreover, 1 falcon tested positive to *M. avium. paratuberculosis* in tissue samples by PCR. In all cases, blood and fecal samples tested negative. In the acid-fast stain, all samples showed the for mycobacteriosis typical rods. Moreover, in 13 samples *Acinetobacter baumannii* was detected by PCR and proven by DNA sequencing. Clinical features included highly elevated WBCs, heterophilia, lymphocytopenia, monocytosis, severe anemia and weight loss. *A. baumannii*, a gram-negative bacillus with the ability to integrate foreign DNA, has emerged as one of the major multidrug resistant bacteria. In veterinary medicine, it has so far been detected in dogs, cats, horses and wild birds. To the authors' knowledge, this is the first report of an *A. baumannii* infection in falcons and of a veterinary *Mycobacterium-Acinetobacter* coinfection.

## 1. Introduction

The genus *Acinetobacter* comprises strictly aerobic, gramnegative and nonfermentative bacteria belonging to the family Moxarellaceae. They are widely distributed in the environment as well as in hospitals, where they can survive on moist or dry surfaces for long periods of time [1] on dry surfaces [2]. *Acinetobacter* can also be found in soil and constitutes as one of the predominant organisms in water [3]. 

*A. baumannii* is the most commonly found *Acinetobacter* species in humans and has emerged as multidrug resistant [1]. Moreover, it has evolved as one of the most important nosocomial pathogens in the past decade [4] particularly in immunosuppressed patients [1]. *A. baumannii* can affect different organs causing pneumonia, meningitis, septicemia and urinary tract [5] and skin infections [4]. However, it is still not entirely clear how *A. baumannii* is transmitted. Its “success” as an emerging pathogen might be based on its ability to integrate large amounts of foreign DNA into its genome, located as significant fractions of ORFs on 28 putative alien islands [5]. Risk factors such as underlying diseases or infections of the affected patient, hospitalization, cephalosporin treatment, and medical procedures may pave the way for *A. baumannii *infections [4]. Antimicrobial treatment can be considered as a predisposing factor for *A. baumannii* infections [4]. Between January 2002 and August 2004, *A. baumannii* infections were diagnosed when “military health officials identified 102 patients with blood cultures that grew *A*. *baumannii* at military medical facilities treating service members injured in Afghanistan and the Iraq/Kuwait region” (CDC, 2004). A potential environmental contamination of traumatic injuries might have been an influencing factor (CDC, 2004). Moreover, *A. baumannii* is known to survive for long periods of time.

In contrast to *A. baumannii* infections, mycobacteriosis is well described in birds and mainly caused by *Mycobacterium avium* and *Mycobacterium genavense*. Both pathogens are excreted with the feces [6, 7] and affect mainly hepatic and gastrointestinal organ systems [8]. Water, soil, plants and other environmental sources serve as reservoirs for the *M. avium* complex [9]. *M. avium* can be transmitted from birds to immunosuppressed humans like AIDS patients [9] and has been isolated from cutaneous tuberculosis lesions in humans [10]. 

General symptoms of mycobacteriosis include weight loss, polyuria, coelomic distention, and diarrhea. Rarely cutaneous masses and lameness can be found [7]. Skin granulomas caused mainly by *M. genavense* can be flaky, dry, or raised ulcers and are usually not painful [11]. Granulomatous dermatitis in psittacines can be caused by *M. genavense*, too [12].

## 2. Material and Methods

### 2.1. Clinical Investigation

From May 2007 to April 2009, a total number of 29 falcons, among them 19 Gyr-Peregrine hybrid falcons, 9 Gyr-Saker hybrid falcons, and 1 peregrine falcon, were presented at the Abu Dhabi Falcon Hospital. 26 falcons were females and 2 Gyr-Peregrine hybrid falcons and 1 Gyr-Saker hybrid falcons were males. All falcons belonged to different owners and were bred by different breeders in UAE and Europe. The falcons' age was between 1 and 3 years and all were used for hunting.

For this study, a group of 18 falcons was selected for which all below-mentioned tests could be performed. For the other 11 falcons, most of the tests were performed but not all tests could be done. Therefore, those falcons were excluded from the study. Out of the 18 falcons, 16 falcons had repeatedly visited the hospital. For 2 falcons, it was the first visit. In most falcons, a time span of at least 3-5 months between being diagnosed without abnormality and the manifestation of visible lesions could be observed. One falcon from the UK came from a breeding center where previously another falcon had been diagnosed with mycobacteriosis granuloma. All 18 falcons were either diagnosed with unspecific symptoms, including anorexia, weight loss, and not flying well or presented only for general examination. However, all falcons had cutaneous yellowish-colored lesions. Those lesions were located on the right and left lower thoracical body wall and abdominal body walls as well as the inside of both thighs. The lesions were opened surgically and tissue material was extracted for examination. Then the lesions were sutured with 3–0 Ethilon* (Ethicon, Johnson and Johnson, Belgium).

### 2.2. Laboratory Diagnostics

#### 2.2.1. Blood Examination

Blood samples for hematological and biochemical examination were taken from the left or right V. cutanea ulnaris superficialis and processed within maximum 30 minutes in the laboratory. The blood hematology was performed according to the protocol of Muller et al. [13]. The ACE-Wasserman biochemistry analyser (Schiaparelli Biosystems) was used to measure the blood chemistry parameters by photometry. In 10 falcons, blood was taken 1–3 times. In 8 falcons, the blood sampling was repeated 3 up to 8 times every 3 to 8 days. The blood sampling of one Gyr-peregrine hybrid falcon was not possible due to poor condition of the bird.

#### 2.2.2. Acid-Fast Bacilli Staining

Acid-fast bacilli staining with Ziehl-Neelsen (ZN) stain (ZN-TB Stain Kit (AFB Stain), GCC Diagnostics, Flintshire, UK) was performed for the biopsied tissues and fecal samples of all falcons tested.

#### 2.2.3. Molecular Analysis

The tissues as well as blood and fecal samples were examined by PCR. DNA was extracted from fresh tissue and one blood sample using DNA-Sorb-A DNA Extraction Kit (Sacace Biotechnologies Srl, Caserta, Italy) as per the manufacturers' instruction followed by amplification of the *Mycobacterium *genus DNA using the *Mycobacterium *genus PCR Kit including 4 different primer pairs for *M. genus, M. tuberculosis, M. avium paratuberculosis,* and* M. avium avium *(Genekam Biotechnology, AG, Germany). The PCR was performed in a final volume of 20 *μ*L. The reaction mixture was then subjected to 38 cycles of amplification. (Denaturation at 94°C for 30 seconds, annealing at 65°C for 60 seconds, and extension at 72°C for 120 seconds). After PCR amplification, a 10 *μ*L aliquot of the PCR products was electrophoresed for 1 hour through a 2% agarose gel. The target band of 236 bp (of *Mycobacterium *genus DNA) was visualized under UV illumination. 

In order to clarify why the *Mycobacterium*-specific PCR was negative in most samples despite a clinically suspected mycobacteriosis and to identify the mycobacterial species, a seminested PCR designed for diagnosis of avian mycobacteriosis [14] was performed with all samples. In brief, for the first round primers 246 and 247 were used to amplify an approximately 600 bp, and the amplicon was then subjected to a second round using the p7 and the 247 primers and resulting in a ~550 bp fragment of the first amplicon. Amplification of the respective fragments was visualized by ethidium-bromide in a 2% agarose gel electrophoresis. For identification, the resulting amplicons were subjected to DNA sequencing. Sequencing was carried out in a 310 ABI PRISM automated sequencer (Applied Biosystems, Langen, Germany) and sequences were compared to sequences available at GenBank using BLAST search [15]. Moreover, samples (except samples 3 and 10, from which no material was left) were screened with a universal bacterial PCR using the 16S_for and 16S_rev primers [16] and as sequencing of the respective amplicons revealed highest identity of the sequences to *Acinetobacter baumannii*, all positive samples were screened with a PCR specific for *A. baumannii* [17] amplifying the internal transcribed spacer (ITS) region. Again, amplification was visualized by 2% agarose gelelectrophoresis and amplicons were excised from the gels and subjected to DNA sequencing.

## 3. Results

### 3.1. Clinical Inspection

Out of 18 falcons, a total number of 26 cutaneous lesions were found. 94% of the falcons had a lesion on the left and/or right body wall, whereas 50% showed lesions on either or both thigh inside areas (Table 1). Except for two falcons treated, all other falcons died or were euthanized as per request of their owners.

In 39% of the falcons, no other microorganisms were detected. In the remaining 61%, more than one infection was found. Among these, 37% of falcons were identified with aspergillosis. 6% showed hepatopathic lesions and in 18%, hepatomegaly was detected. Moreover, 6% suffered from pseudomoniasis. Airsacculitis was detected in 6% and infestation with *Serratospiculum seurati* worms in another 6% of falcons.

### 3.2. Blood Examination

The blood examinations were separated according to the hybrid species. Moreover, the mean values for hematology and blood biochemistry of all falcons in the study were established. Furthermore, the blood parameters' mean values of falcons with mycobacteriosis combined with *A. baumannii* infection were evaluated (Tables 2 and 3). The established values were compared to reference values for hematology [13, 18] and biochemistry [18].

### 3.3. Ziehl-Neelsen Staining

**Figure 1:**
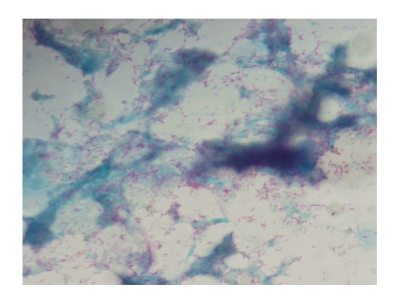
Acid fast organisms stain in bright pink color.

In the Ziehl-Neelsen (ZN) stain, acid-fast bacilli were detected in the cytological squash of biopsied cutaneous tissues as well as fecal material of all falcons tested. In 50 %, the stain revealed acid-fast bacilli covering 2/3 or more of each examined microscopic field (Figure 1). In the remaining cases, 1/3 to 2/3 of the microscopic slides were covered with acid-fast bacilli in the ZN stain. Only in 11%, acid-fast bacilli were additionally identified in the feces material.

### 3.4. Molecular Results

Molecular results are summarised in Table 4. In the *Mycobacterium* genus PCR, all samples except samples 8, 9, 10, and 11 were positive. The *Mycobacterium avium* subspecies* paratuberculosis* PCR was positive in one case (sample 3). Samples were then also screened with a seminested diagnostic PCR for avian mycobacteriosis. However, unfortunately not of all samples enough material was left, so this PCR could only be conducted with 20 samples as sample 9, and 18 was tested twice. Of these, 80% tested positive in the seminested PCR. Sequencing of the amplicons revealed 100% sequence identity to the *M. avium* complex (MOC) in all positive cases. Moreover, 60% were positive in the specific *A. baumannii* PCR.

### 3.5. Therapeutic Results

Both falcons treated were administered by a monotherapy of rifampicin 30 mg/kg once a day. One falcon was treated for 68 days and recovered. Subsequent PCR tests for *Mycobacterium *sp. were negative. The other falcon was treated for 45 days. It returned after 39 days in apparently good condition and without visible granulomatous lesions with intralesional mycobacteria, however, no further PCR testing could be performed.

## 4. Discussion

Between May 2007 and April 2009, mycobacteriosis was detected in 18 falcons. 16 of them tested positive in the seminested mycobacteria PCR. Moreover, the 2 negative samples tested positive for *M.* genus PCR. Furthermore, 2 samples were additionally tested positive for *M. avium* and one for *M. avium paratuberculosis*. These findings stand in sharp contrast to a study on pet birds where the causative agents were mainly *M. genavense* and only in one case *M. avium* [14] In the vast majority of cases (16 falcons), the time frame of the examination of clinically healthy falcons until clinical manifestation of mycobacteriosis ranged between 3 and 5 months. In two cases, falcons were diagnosed with mycobacteriosis at the time of their first presentation at the Abu Dhabi Falcon Hospital. Therefore, the time of disease manifestation is not known. One of these two falcons was also tested positive for *A. baumannii.*

Cutaneous lesions of the diseased falcons were found only as localized lesions on both body walls ranging from the lower thoracical part to the abdominal areas as well as on the inside of left and right thighs. The sizes were variable and ranged from 0.5 mm up to 5 cm. The yellowish lesions were of firm, leathery-like consistency. In contrast, in quails firm, pale-colored nodes of different sizes could be found from the midcervical region up to the thoracic inlet [19].

Mycobacteriosis cannot be easily identified by hematological changes or clinical symptoms until the disease reaches more advanced stages [8]. In our study, the hematological changes showed unified patterns with strong anemia, high white blood cell counts, heterophilia and strong lymphocytopenia. Furthermore, basophilia was present. Further hematological changes indicating a mycobacteriosis are presence of low albumin and low total protein levels [20], which however, could not be supported by the findings of our study. In our study, albumin and total protein levels were slightly elevated. On the other hand, changes in hematological parameters have been described as nonspecific [21]. In falcons, severe bacterial of fungal infections like aspergillosis and pseudomoniasis can cause highly elevated WBC and heterophilia associated with lymphocytopenia. As in our study 39% of the falcons were not diagnosed with any other disease than mycobacteriosis, the blood parameter changes could not be caused by another underlying disease. However, the hematological blood picture of the diseased falcons in our study showed very distinctive change patterns, thus, indicating a mycobacteriosis. In all performed acid-fast bacilli stains, bacilli were clearly identified which is highly indicative for a mycobacteriosis and got confirmed through the PCR results. 

A difficulty in avian mycobacteriosis identification is the fact that the bacterium gets mainly shed in the advanced stages of the disease, but not in the early ones [22]. Unsuccessful identification of mycobacterial species despite repeated trials are reported in research [14]. This problem might be related to severe DNA damage during processing [23] which could be an explanation for the difficulties of mycobacterium detection in our study. Another explanation for unsuccessful mycobacterial detection might be the extremely low quantity of mycobacterial DNA in paraffin-embedded tissues [23]. Due to the unequal distribution of mycobacteria in tissue samples, this reason might be also applicable to the tissue samples in our study although only fresh and not paraffin-embedded tissues were examined.

Treatment of mycobacteriosis can take up to one year and in successful cases included a combination of different drugs like clarithromycin, ethambutol, rifabutin, and enrofloxacin [7]. Rifampicin is highly efficient in combination with other antimicrobials. It can be used as monotherapy although during the treatment period limitations of its efficiency may be caused through resistances [24]. In our case, rifampicin was used as monotherapy for 45 and 68 days leading to the survival of the two only falcons treated. One of them was tested negative repeatedly. Moreover, its skin lesion disappeared completely after gradual regression. At the time of treatment, the diagnosis of *A. baumannii* infection was not yet established.

The finding of the presence of *A*. *baumannii* was completely unexpected. So far, as to the authors' knowledge, no cutaneous mycobacteriosis lesions in animals and birds have been found in association with *A. baumannii* so far. However, *A. baumannii* infections cannot only be observed in humans, but also in animals like cats, dogs [25], and horses [26]. Random sampling of a zoological collection in Japan led to the isolation of *Acinetobacter baumannii* from the feces of wild birds. However, it was not mentioned if any pathological condition of the birds was present [27]. Commercially available microbiology testing systems like Mini-API or Vitek are not successful in the detection of *A. baumannii* but its identification by ITS sequencing is regarded as a very reliable method [17]. A 2.6 kbp DNA fragment was identified from a multidrug-resistant *A. baumannii* strain in a horse. This cassette structure was identical to that isolated in an unrelated human *Acinetobacter* strain. As the numbers of isolates in humans carrying the class 1 integrons are on the rise, this might be applicable to animals as well. Therefore, the transmission from human-to-animal or animal-to-human of MDR *Acinetobacter *spp. seems to be possible [28]. 

In the regular monthly facility screening of the Abu Dhabi Falcon Hospital, *A. baumannii* was never detected throughout the years 2006, 2007, and 2008—except in December 2007. In the facility screening of December 29th 2007, *A. baumannii* was identified on the exam room work table, adjacent work bench and computer as well as one glove used for holding falcons. This clearly indicates that this pathogen was not present in the hospital 17 month before the diseased falcons were diagnosed. Moreover, after immediate cleaning up of the affected areas, *A. baumannii* was not detected again until August 2008. Due to the fact that one falcon of this study was presented in the Abu Dhabi Falcon Hospital for examination on December 27th, 2007, these findings strongly indicate that the infected falcon had shed the bacteria when arriving at the hospital. The contamination came therefore presumably through handling of this falcon. Moreover, in one falcon that was presented for the first time at the Abu Dhabi Falcon Hospital, *A. baumannii* was isolated from a cutaneous lesion. This leads to the assumption that a nosocomial infection of the falcons in our study does not seem likely although nosocomial *A. baumannii* infections are frequently found in hospitals [4]. The route of transmission of mycobacteriosis and *A. baumannii* in the falcons of this study remains unclear due to the fact that they come from different breeders, owners and are kept in different places.

It seems likely that the falcons became infected with *A. baumannii* through contaminated wild birds, possibly through their feces [27], which they might have caught while hunting. However, it remains unclear to what extent wild birds are infected with *A. baumannii *and what might be the likelihood of a possible interspecies transmission. Further research is needed in this subject. Moreover, the findings of this study raise the question if a human-to-birds or birds-to-human transmission of *A. baumannii* exists which requires further investigation. 

## Figures and Tables

**Table 1:** Location and diameter of cutaneous lesions.

S. No	Lesion on left lower thoracical and abdominal wall	Lesion on right lower thoracical and abdominal wall	Lesion on medial part of left thigh	Lesion on medial part of right thigh
1	0.5–1 cm	0.5–1 cm		
2			2.1–5 cm	
3	0.5–1 cm	0.5–1 cm	1.1–2 cm	1.1–2 cm
4	1.1–2 cm			
5		larger than 5 cm		larger than 5 cm
6	2.1–5 cm			
7	2.1–5 cm			
8			1.1–2 cm	
9				0.5–1 cm
10	1.1–2 cm			
11	2.1–5 cm		2.1–5 cm	
12	2.1–5 cm	2.1–5 cm		
13	2.1–5 cm			
14	2.1–5 cm			
15			1.1–2 cm	
16				1.1–2 cm
17	2.1–5 cm	2.1–5 cm		
18		larger than 5 cm		

**Table 2:** Mean of hematology results of Gyr-Saker and Gyr-Peregrine hybrid falcons tested positive for *A. baumannii* and mycobacteriosis.

Parameters	Gyr-Saker with Mycobacteriosis	Gyr-Saker reference values	Gyr-Peregrine with A. baumannii + Mycobacteriosis	Gyr-Peregrine reference values
	*n* = 2		*n* = 9	
RBC (×10^12^/l)	2.15	2.18–2.48	2.02	2.13–2.65
Hb (g/dl)	12.60	16.23–19.23	12.23	16.33–19.47
Hct %	38.10	46.91–56.23	34.10	47.25–56.75
MCV (fl)	166.45	200.29–243.49	186.62	194.32–245.08
MCH (pg)	58.40	69.41–83.07	67.48	67.14–84.04
MCHC (g/dl)	35.10	32.85–35.99	36.51	32.95–35.91
WBC (×10^9^/l)	29.70	5.28–9.72	31.53	5.28–9.82
Heterophils%	71.00	46.02–53.78	72.90	46.41–53.41
Lymphocytes%	19.90	41.20–47.02	22.14	40.82–47.54
Monocytes%	3.00	2.84–6.00	3.31	2.77–6.03
Eosinophils%	1.00	0.37–2.21	1.26	0.29–2.45
Basophils%	0.50	0.25–0.55	0.56	0-0

**Table 3:** Mean of blood biochemistry results of Gyr-Saker and Gyr-Peregrine hybrid falcons tested positive for *A. baumannii* and mycobacteriosis.

Parameters	Gyr-Sakerwith A. baumannii + Mycobacteriosis	Gyr-Saker reference values	Gyr-Peregrinewith A. baumannii + Mycobacteriosis	Gyr-Peregrine reference values
	*n* = 2		*n* = 9	
GGT (U/L)	13.6	5.5–11.5	10.3	6.0–12.0
AST (U/L)	103.9	45.0–122.3	138.1	47.0–110.0
ALT (U/L)	122.8	37.2–91.0	116.6	44.9–73.5
TP (g/dl)	3.7	2.2–3.4	3.4	2.15–3.35
ALB (g/dl)	1.3	0.9–1.2	1.1	0.73–1.35
LDH (U/L)	2825.9	788.8–1493.3	2811.5	802.0–1457.0
CK (U/L)	466.9	312.3–681.3	740	341.0–668.7
UA (mg/ml)	5.9	3.4–7.5	9.5	3.60–7.0
UREA (mg/ml)	17.6	6.4–20.1	29.1	7.0–17.0
CHOL (mg/ml)	105.3	159.0–267.0	144.8	167.0–267.0
ALP (U/L)	227.8	—	169.2	—
AMYL (U/L)	159.5	—	857.6	—

**Table 4:** Diagnostic results with Polymerase Chain reaction of positive cases in the second test run.

S. No.	Mycobacterium genus	Mycobacteria seminested	Mycobacterium avium avium	Mycobacterium avium tuberculosis	Acinetobacter baumannii
1	+	+	−	−	+
2	+	+			+
3	+	+	No sample	+	+
4	+	+	+	−	−
5	+	+	−		+
6	+	+			−
7	+	+	+	−	−
8	_	+	No sample		−
9	−	+	−		+
10	−	+			−
11	−	+			+
12	+	−			+
13	+	+			+
14	+	+			+
15	+	−		+	−
16	+	+			+
17	+	+			+
18	+	+			+
